# Theoretical investigation on $\hbox {BeN}_{{2}}$ monolayer for an efficient bifunctional water splitting catalyst

**DOI:** 10.1038/s41598-020-77999-8

**Published:** 2020-12-08

**Authors:** M. R. Ashwin Kishore, R. Varunaa, Amirhossein Bayani, Karin Larsson

**Affiliations:** 1 Department of Chemistry, Ångström Laboratory, Uppsala University, Box 538, 751 21 Uppsala, Sweden; 2 Department of Physics, Central University of Tamil Nadu, Thiruvarur, Tamil Nadu 610101 India

**Keywords:** Materials science, Materials for energy and catalysis, Nanoscale materials, Theory and computation

## Abstract

The search for an active, stable, and abundant semiconductor-based bifunctional catalysts for solar hydrogen production will make a substantial impact on the sustainable development of the society that does not rely on fossil reserves. The photocatalytic water splitting mechanism on a $\hbox {BeN}_{{2}}$ monolayer has here been investigated by using state-of-the-art density functional theory calculations. For all possible reaction intermediates, the calculated changes in Gibbs free energy showed that the oxygen evolution reaction will occur at, and above, the potential of 2.06 V (against the NHE) as all elementary steps are exergonic. In the case of the hydrogen evolution reaction, a potential of 0.52 V, or above, was required to make the reaction take place spontaneously. Interestingly, the calculated valence band edge and conduction band edge positions for a $\hbox {BeN}_{{2}}$ monolayer are located at the potential of 2.60 V and 0.56 V, respectively. This indicates that the photo-generated holes in the valence band can oxidize water to oxygen, and the photo-generated electrons in the conduction band can spontaneously reduce water to hydrogen. Hence, the results from the present theoretical investigation show that the $\hbox {BeN}_{{2}}$ monolayer is an efficient bifunctional water-splitting catalyst, without the need for any co-catalyst.

## Introduction

Fossil resources, such as oil, gas, and coal are depleting rapidly, which urge the research community to find alternative energy technologies to be able to solve the demands of global energy issues. One of the promising alternative technologies is solar hydrogen production through water splitting with the help of semiconductors. The past few decades have seen tremendous efforts in the discovery and fabrication of highly efficient semiconducting photocatalysts for water splitting, including metal oxides, metal sulfides, metal nitrides, and metal oxynitrides^1–5^. However, some of these materials suffer from poor absorption of solar light, and photo-corrosion. Therefore, finding an efficient photocatalyst for water splitting is still a challenging problem. At present, the best-known catalyst for oxygen evolution reaction (OER) and hydrogen evolution reaction (HER) is ruthenium oxides, iridium oxides, and platinum-based materials^6–9^. However, the scarcity and high cost of noble metals and precious metal oxides impose severe limitations for large-scale applications. The development of efficient and stable materials for OER and HER, by modifying chemical composition, structure, morphology, and support, is therefore of largest importance.

Two-dimensional (2D) materials have attracted increasing attention in numerous research fields during the past few years. This is owing to their superior properties, which are completely different from their bulk counterparts. The prospect of using these 2D materials in the field of energy conversion and storage, is very promising due to their various intrinsic properties, such as the presence of many surface-active sites, enhanced electron-hole separation, fast mobility of photogenerated charge carriers, and reduced recombination rate. After the graphene’s breakthrough, a wide range of 2D materials for HER and OER have been extensively examined^10–14^. Graphene-based materials, like N-doped graphene^15,16^ and N-containing co-doped graphene^17^, have been reported as excellent catalysts for HER and OER of high activity. Transition metal dichalcogenides (TMDs)^18,19^, metal monochalcogenides^20^, MXenes^21,22^, g-$\hbox {C}_{{3}}\,\hbox {N}_{{4}}$^23,24^, and $\hbox {C}_{{2}}$N^25–27^, have also been identified as potential catalysts for water splitting.

The $\hbox {BeN}_{{2}}$ monolayer is a graphene-like honeycomb lattice with an optically active direct bandgap of 2.23 eV^28^. This is a theoretically predicted material that possesses high mechanical, thermal, and lattice dynamical stability. The reported carrier mobility at room temperature is higher than that of many other 2D materials^29–31^. All these exceptional properties make the $\hbox {BeN}_{{2}}$ monolayer very attractive for photocatalytic applications. Very few reports are available on this material for energy storage and conversion applications to date. Ding et al.^32^ elucidated the oxygen reduction reaction mechanism for the $\hbox {BeN}_{{2}}$ monolayer. It was found that the overpotential is 0.45 eV in an acidic environment, and with zero overpotential in an alkaline environment. Moreover, Wei et al.^33^ studied the photocatalytic water splitting activity onto this monolayer through the band edge position with respect to the water redox potentials. However, the reaction mechanism for the catalysis of HER and OER have not yet been reported for this material.

The purpose with the present study has been to get a deeper understanding about the reaction mechanisms of HER and OER that takes place on a $\hbox {BeN}_{{2}}$ monolayer, and also to evaluate their overpotentials. These HER and OER catalytic processes have therefore been systematically investigated, using calculations based on the density functional theory (DFT). As a result, the calculated electronic properties, and band edge alignment, indeed show that the $\hbox {BeN}_{{2}}$ monolayer has the potential to function as a bifunctional catalyst in the splitting of water by using solar light. Moreover, the investigation of OER and HER mechanisms resulted in overpotentials of 0.83 V and 0.52 V (with reference to NHE), respectively. These very positive theoretical results indeed spread light on further experimental explorations.

## Results and discussion

### Structural and electronic properties

**Figure 1:**
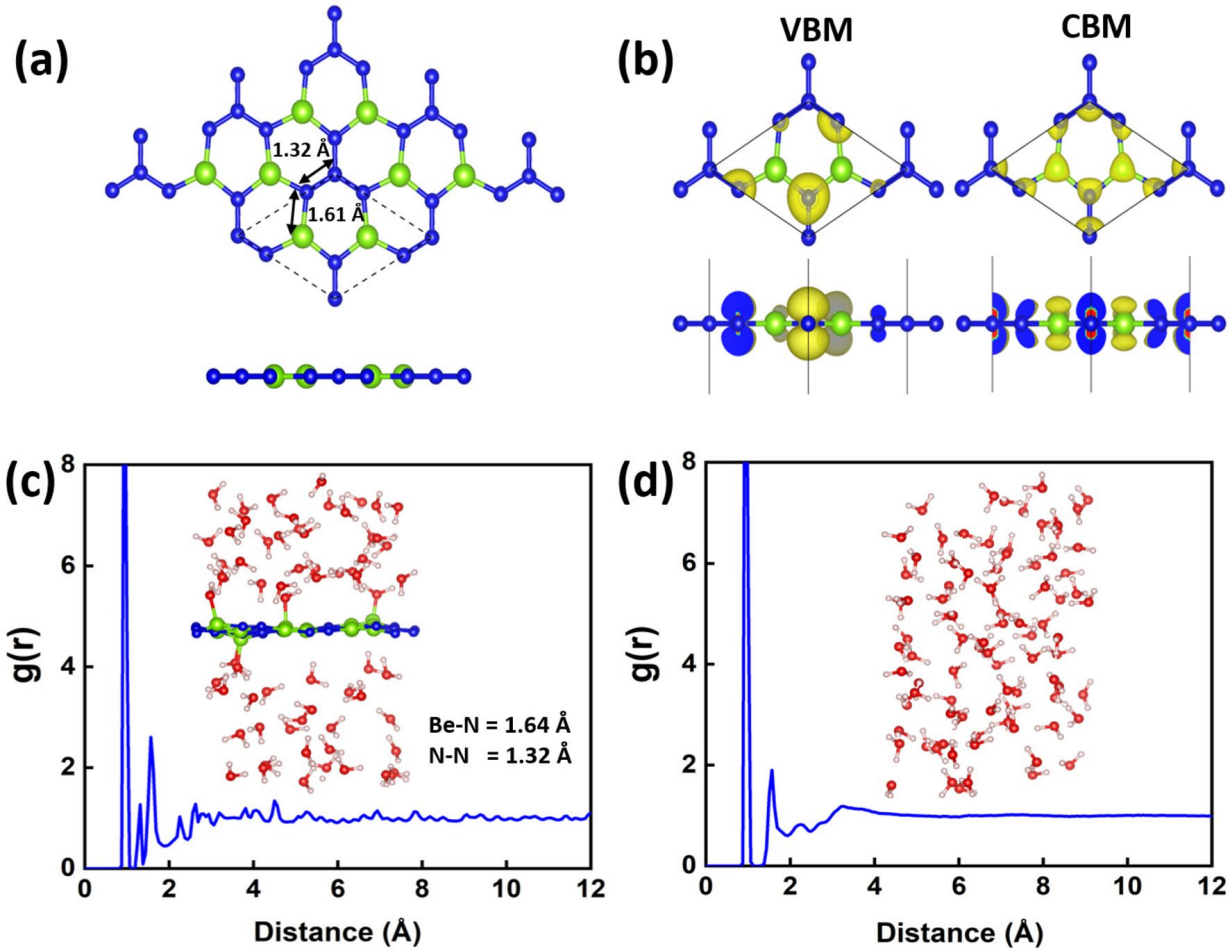
This figure shows some results for a monolayer of $\hbox {BeN}_{{2}}$: (**a**) Schematic geometrical structure in top and side views (the dashed lines indicate the unit cell); (**b**) Top and side views of isosurface plots of the charge densities at the VBM and CBM, respectively (the isosurface value is 0.008 e Å$^{-3}$); The normalized pair correlation functions g(r) of (**c**) $\hbox {BeN}_{{2}}$ monolayer in liquid water and (**d**) liquid water after annealing at 300 K for 5 ps. The inset in (**c**) shows the structural evolution of the $\hbox {BeN}_{{2}}$ in liquid water and average bond lengths of Be-N/N-N. The inset in (**d**) shows the structural evolution of water molecules after annealing at 300 K for 5 ps, respectively. Be, N, O, and H atoms are marked in green, blue, red, and beige, respectively.

The optimized structure of the $\hbox {BeN}_{{2}}$ monolayer is shown in Fig. 1a. It possesses the P-62 M space group with a point group of $\hbox {D}_{{3h}}$. The calculated lattice constant (a=4.54 Å ), bond lengths (Be−N = 1.61 Å ; N−N = 1.32 Å ), and bond angles (Be−N−Be = 108.3° ; Be−N−N = 125.8°) are in accordance with previous reports^28,33^. Moreover, the band decomposed charge density, corresponding to the valence band maxima (VBM) and the conduction band minima (CBM) at the $\Gamma$ point, is displayed in Fig. 1b. As can be seen in Fig. 1b, the charges at the VBM comprise the N-$\hbox {p}_{{z}}$ states, and that the charges at CBM arise from hybridization of both Be and N-$\hbox {p}_{{z}}$ states. The cohesive energy were calculated to evaluate the stability of the $\hbox {BeN}_{{2}}$. The cohesive energy is defined as:

1 $$\begin{aligned} E_{coh} = (nE_{Be} + 2nE_{N} - E_{BeN_{2}})/3n \end{aligned}$$

where $E_{Be}, E_{N}, and\, E_{BeN_{2}}$ are the total energy of single Be atom, single N atom and $\hbox {BeN}_{{2}}$ sheet, respectively. n is the number of formula unit in the $\hbox {BeN}_{{2}}$ cell. The calculated cohesive energy of $\hbox {BeN}_{{2}}$ is 5.03 eV/atom which is larger than other well known 2D materials such as silicene (4.57)^34^, phosphorene (3.48)^35^, and $\hbox {Al}_{{2}}$C sheet (4.58)^36^. This indicates the bonding in $\hbox {BeN}_{{2}}$ is strong and stable.

In order to gain more insight into the thermal and dynamical stability of the $\hbox {BeN}_{{2}}$ monolayer, analyses of phonon dispersion spectra, as well as of $ab-initio$ molecular dynamics simulations, have been performed. As shown in Fig. S1 (in the Supplementary file), it is evident that $\hbox {BeN}_{{2}}$ monolayer is dynamically stable since all vibration modes are real, and there are no imaginary frequency modes in the entire Brillouin zone. Moreover, the total potential energy fluctuation is minimum throughout the simulation period (as shown in Fig. S2). This indicates that the structure is not changed (with time) at room temperature. Also, the inset shows that the structure of the $\hbox {BeN}_{{2}}$ monolayer, at the end of the simulation, is planar with little buckling. It does not suffer drastic structural distortion or transformation, and, hence, this material is stable at room temperature.

**Figure 2:**
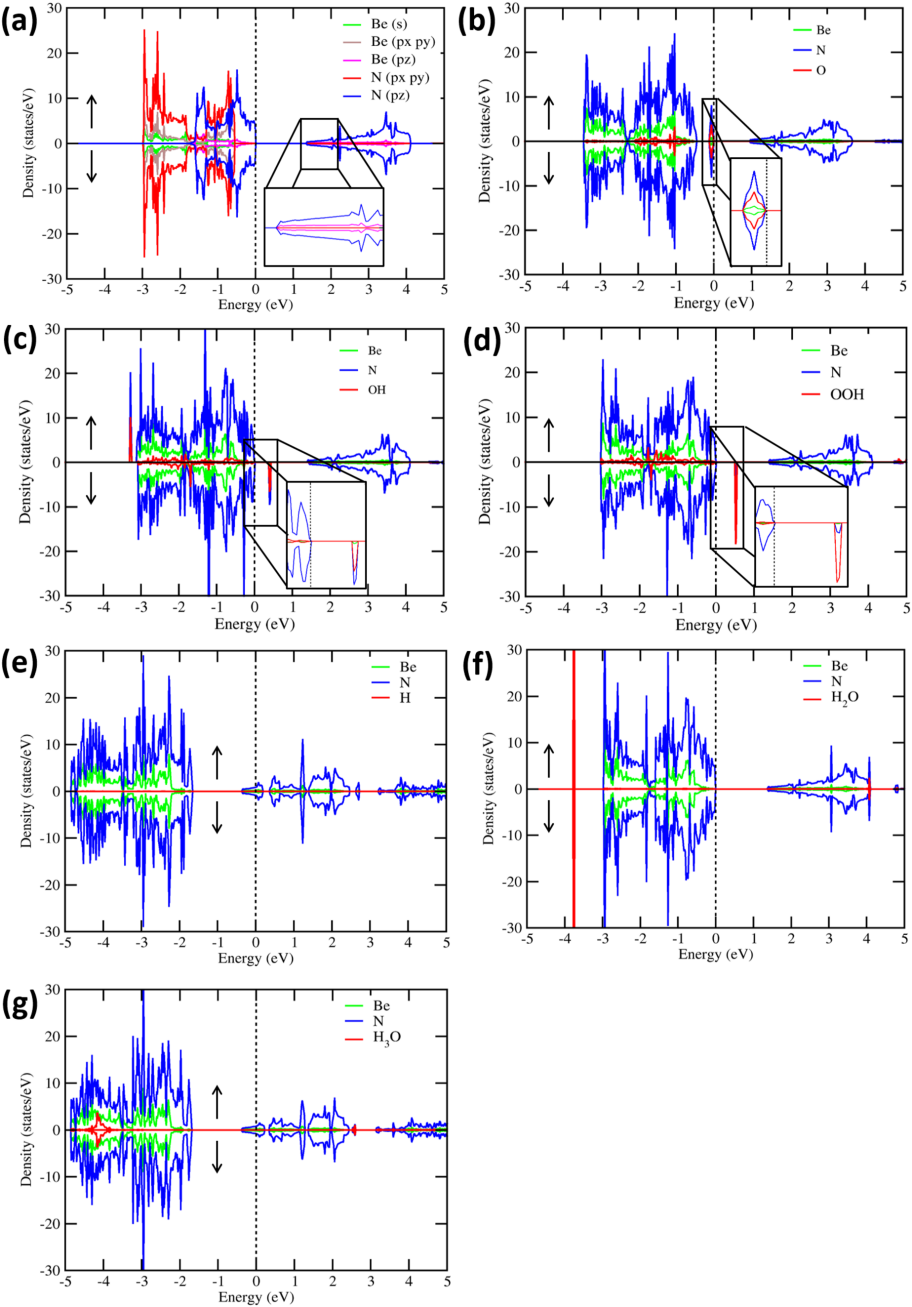
Calculated partial density of states for (**a**) a $\hbox {BeN}_{{2}}$ monolayer, and for the (**b**) O, (**c**) OH, (**d**) OOH, (**e**) H, (**f**) $\hbox {H}_{{2}}$O, and (g) $\hbox {H}_{{3}}$O adsorbates onto the $\hbox {BeN}_{{2}}$ monolayer. Arrows indicate the up ($\uparrow$) and down ($\downarrow$) spin channels. A magnified view of the states are shown in the insets.

It is also essential to investigate the stability of the $\hbox {BeN}_{{2}}$ monolayer in an aqueous environment. Therefore, the $\hbox {BeN}_{{2}}$ monolayer was placed in liquid water with a fixed density of 1 g cm$^{-3}$ and annealed at 300 K for five pico-seconds. The calculated normalized pair correlation functions show some sharp peaks in the long-range part between 7 and 12 Å  (see Fig. 1c), and this indicates that the $\hbox {BeN}_{{2}}$ monolayer remains crystalline after the annealing step in an aqueous environment. This is due to the fact that crystalline materials show long-range order features, and the amorphous materials show long range disorder features. The inset shows the structural evolution of the $\hbox {BeN}_{{2}}$ in liquid water, and is buckled due to interaction with water molecules. 2D materials usually show wrinkles at infinite temperatures, and these are reasons why the peaks are small. The calculated normalized pair correlation function for $\hbox {H}_{{2}}$O shown in Fig. 1d indicates the liquid nature of water, which is consistent with the XRD measurements^37^.

Next, to evaluate the corrosion resistance or electrochemical stability of $\hbox {BeN}_{{2}}$, Pourbaix diagram is predicted. Figure S3 (in the supplementary information) shows the Pourbaix diagram of the $\hbox {BeN}_{{2}}$ system. An ionic concentration of 10$^{-2}$ M for both Be and N species is used for the analysis. Materials with Gibbs free energy of decomposition $\Delta \,\hbox {G}_{{pbx}}$ = 0 eV/atom are considered to be stable, whereas, for $\Delta \,\hbox {G}_{{pbx}}$ > 0, they decompose to Pourbaix-stable phases in thermodynamic equilibrium. In general, materials which decompose to at least one solid phase might form a passivation layer on the surface to prevent further corrosion. As can be seen in Fig. S3, $\hbox {BeN}_{{2}}$ has a large window of thermodynamic stability in the whole alkaline region and some parts of the acidic region. However, at pH 0 in the redox region, the minimum of the $\Delta \,\hbox {G}_{{pbx}}$ is found to be 0.24 eV/atom. Earlier reports have shown that the well-known semiconducting materials such as GaP, Zn ($\hbox {FeO}_{{2}}$)$_{2}$, Si, $\hbox {WSe}_{{2}}$, GaAs, $\hbox {Fe}_{{2}}\,\hbox {O}_{{3}}$, $\hbox {WO}_{{3}}$, and $\hbox {TiO}_{{2}}$ are found to be stable experimentally, have a $\Delta \,\hbox {G}_{{pbx}}$ as high as 0.5 eV/atom. These materials are stable against corrosion due to self-passivation and formation of more stable solid-state surface phases^38^. Therefore, the $\hbox {BeN}_{{2}}$ at pH 0 can be considered to be stable in water through the development of a passivating film.

The electronic properties of the $\hbox {BeN}_{{2}}$ monolayer were evaluated by using band structure and density of states (DOS) analysis. The calculated band structure and DOS spectrum, using a GGA-PBE functional, are plotted in Fig. S3a. It is there shown that the $\hbox {BeN}_{{2}}$ monolayer is a direct bandgap semiconductor with a bandgap value of 1.34 eV at the $\Gamma$ point. From the DOS spectrum it is clear that the VBM and CBM are originated from N-p states and hybridization with N and Be-p states, respectively. Be and P states are hybridized over the whole valence band, which indicates the presence of in-plane covalent bonding within the $\hbox {BeN}_{{2}}$ monolayer. The band structure of the $\hbox {BeN}_{{2}}$ monolayer, using the HSE06 functional (see Fig. S3b), was also calculated. The band dispersion is the same as that of PBE band structure, but the calculated HSE06 bandgap value is 2.26 eV. In general, the HSE06 functional improves the bandgap, but it has been shown that the single-shot $\hbox {G}_{{0}}\,\hbox {W}_{{0}}$ calculation can provide an accurate estimation of the electronic bandgap in solids, which is close to experimental values. Therefore, the bandgap has there been calculated by using single-shot $\hbox {G}_{{0}}\,\hbox {W}_{{0}}$ calculation, and the obtained bandgap value for the $\hbox {BeN}_{{2}}$ monolayer is 3.16 eV. This bandgap value was thereafter used to align the band edge positions with respect to water redox potentials (which will be discussed below).

**Figure 3:**
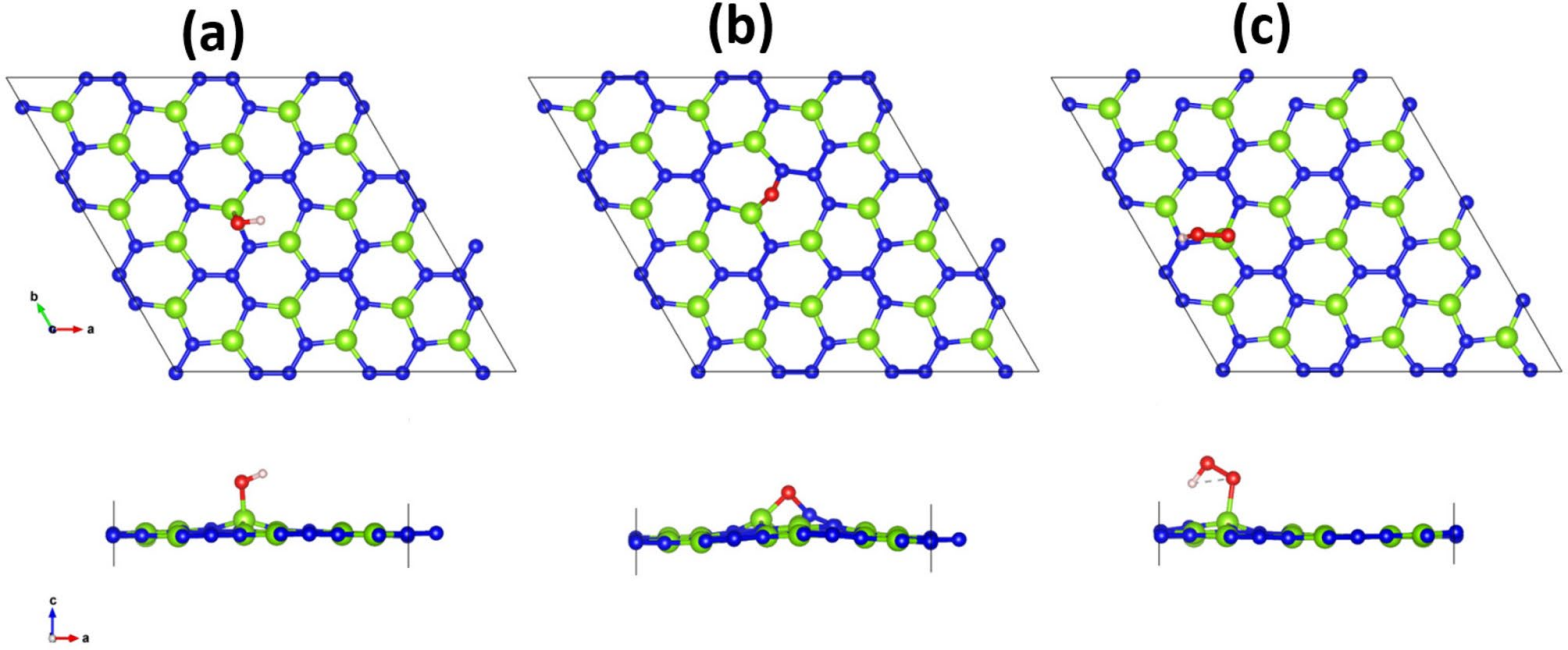
Optimized geometries of possible intermediates in the oxygen evolution reaction; (**a**) *OH, (**b**) *O, and (**c**) *OOH.

Before investigating the OER and HER mechanisms, the adsorption of the reaction intermediates O, OH, OOH, H, $\hbox {H}_{{2}}$O, and $\hbox {H}_{{3}}$O$^{+}$ onto the $\hbox {BeN}_{{2}}$ surface was studied. To understand the charge transfer characteristics between the reaction intermediates and $\hbox {BeN}_{{2}}$ surface, the charge density difference $\Delta \rho$(r) was calculated (see Eq. (1))

2 $$\begin{aligned} \Delta \rho (r) = \Delta \rho _{BeN_{2}+Mol(r)} - \Delta \rho _{BeN_{2}} - \Delta \rho _{Mol(r)} \end{aligned}$$

where $\Delta \rho _{BeN_{2}+Mol(r)}$, $\Delta \rho _{BeN_{2}}$, and $\Delta \rho _{Mol(r)}$ are the charge densities of the adsorbed system, isolated $\hbox {BeN}_{{2}}$ sheet, and isolated molecule in adsorbing configuration, respectively. Bader’s atoms-in-molecule approach is used to measure the charge being transferred quantitatively^39^. For the situation with an atomic O adsorbate, the oxygen atom was found to break the Be−N bond and form a chemical bond with both Be and N. As can be seen in Fig. 3b, the resulting bond lengths are 1.55 Å (Be−O) and 1.37 Å (O−N). These values can be understood from the calculated partial density of states (PDOS) (as displayed in Fig. 2a,b). For the $\hbox {BeN}_{{2}}$ monolayer, it is obvious that the VBM originates from N-$\hbox {p}_{{z}}$ and the CBM originates from N-$\hbox {p}_{{z}}$ (with a small contribution from Be-$\hbox {p}_{{z}}$). In addition, the $\hbox {p}_{{x}}$ and $\hbox {p}_{{y}}$ orbitals of the Be and N atoms are involved in forming the in-plane covalent bonds, which indicates that this specific material is sp$^{2}$ hybridized. Moreover, the weak N($\hbox {p}_{{z}}$)-Be($\hbox {p}_{{z}}$) overlap prone to react with the incoming O atom. It is evident from the PDOS of the O adsorbed system that the O atom will strongly bind with N and Be as their *p*-states are strongly hybridized near the Fermi level. Due to this circumstance, the Be and N atoms will buckle up from its *xy*−plane, which is a clear indication of a sp$^{3}$ bonding feature. Futhermore, it can be seen from the iso-surface of charge density difference plot, as shown in Fig. S4a, that the charges will be transferred from the $\hbox {BeN}_{{2}}$ monolayer to the adsorbed O species. The Bader charge analysis indicated that the amount of charge transferred from the $\hbox {BeN}_{{2}}$ monolayer to the O species is 0.83 $\textit{e}$.

For the situation with an OH adsorbate, it was shown to prefer to bind on top of the Be atom, and the Be−O bonding distance was found to be 1.63 Å . The Be atom is thereby somewhat buckled up from its *xy*−plane, indicating the occurrence of sp$^{3}$ hybridization. This has also earlier been observed for OH adsorption onto 2D boron-nitride and graphene^40^. Moreover, the OH adsorption also introduced unoccupied defect states in the bandgap region, as can be seen from the PDOS spectrum in Fig. 2c. These states were observed to be hybridized with the N/Be-$\hbox {p}_{{z}}$ states in the valence band, which indicates a strong interaction between the OH radicals and the $\hbox {BeN}_{{2}}$ monolayer. In addition, the charge transfer analysis (see Fig. S4b) indicates that charges are depleted from $\hbox {BeN}_{{2}}$ monolayer and accumulated on the OH adsorbate. The following Bader charge analysis indicates that 0.53 $\textit{e}$ has been transferred upon OH adsorption.

As can be seen in Fig. 3c, OOH adsorbate prefers to bind on top of a surface Be atom, and the calculated bond lengths are 1.79 Å, 1.36 Å, and 1.03 Å for Be−O, O−O, and O−H, respectively. As was the situation with the OH adsorbate, the OOH adsorbate introduces unoccupied defect states in the bandgap region, and does also hybridize with N/Be-$\hbox {p}_{{z}}$ states in the valence band (see Fig. 2d). Upon OOH adsorption, charges will be transferred from $\hbox {BeN}_{{2}}$ surface to OOH (as can be seen in Fig. S4c). The calculated Bader charge that OOH accepts is 0.27 $\textit{e}$. From Bader analysis, it was found that the O adsorbate accepts more charges from $\hbox {BeN}_{{2}}$ layer than OH and OOH adsorbate. This is the reason why the adsorption energy is stronger for atomic O than OH and OOH adsorbate.

Atomic hydrogen was also observed to prefer to adsorb strongly onto N (in $\hbox {BeN}_{{2}}$), with an N−H bond length of 1.03 Å. As can be seen in the PDOS spectrum of the H-adsorbed system (see Fig. 2e), there are no hydrogen induced states within the bandgap. However, the Fermi level is shifted from a position within the bandgap to a position within the conduction band. This shift in Fermi level position is due to the well-known electron donor character of the atomic H. The charge transfer analysis confirms that there is a substantial depletion of electrons in the atomic H upon adsorption (see Fig. S4d). A Bader charge analysis reiterates that 0.44 $\textit{e}$ is depleted from H.

The $\hbox {H}_{{2}}$O molecule was shown to prefer an on top position of the Be atom in ($\hbox {BeN}_{{2}}$), with a distance of 1.94 Å. The PDOS spectrum displayed in Fig. 2f shows that the VBM and CBM of the $\hbox {BeN}_{{2}}$ monolayer are not significantly altered when $\hbox {H}_{{2}}$O is being adsorbed. However, there are $\hbox {H}_{{2}}$O induced states in valence band (VB) and in the conduction band (CB), and they are situated at −3.8 eV (VB) and at 4 eV (CB). A charge transfer analysis indicates that only a tiny amount of charge transfer occurs between the $\hbox {BeN}_{{2}}$ monolayer and the $\hbox {H}_{{2}}$O adsorbate (see Fig. S4e). Moreover, a Bader charge analysis shows that a net charge of 0.01 $\textit{e}$ is transferred from the $\hbox {BeN}_{{2}}$ monolayer to the $\hbox {H}_{{2}}$O adsorbate.

For the situation with $\hbox {H}_{{3}}$O$^{+}$, it was found to dissociatively adsorb onto the $\hbox {BeN}_{{2}}$ surface, forming a non-adsorbed $\hbox {H}_{{2}}$O molecule and an H adsorbate. The H atom will then chemically bind to the surface N atoms, and the water molecule will form a hydrogen bond with the H adsorbate (Fig. 5c). The PDOS spectrum of $\hbox {H}_{{3}}$O$^{+}$ adsorbed system is shown in Fig. 2g, displaying both the atomic H adsorption features, as well as the $\hbox {H}_{{2}}$O molecular feature. As such, the Fermi level is shifted from a position within the bandgap to a position within the conduction band, and there are $\hbox {H}_{{2}}$O induced states within the valence band at around − 4 eV. Moreover, Fig. S4f shows that the charges are redistributed between $\hbox {H}_{{2}}$O and atomic H, with a net charge of 0.49$\textit{e}$ being transferred to the $\hbox {BeN}_{{2}}$ monolayer (mainly from the adsorbed H species). The large transfer of charge from H to N (in $\hbox {BeN}_{{2}}$) will thereby induce a bond breakage within $\hbox {H}_{{3}}$O$^{+}$, leading to the two separate entities; molecular $\hbox {H}_{{2}}$O and the H adsorbate.

### Band edge alignment

The band edge position with respect to water redox potentials is a crucial factor in determining whether a material is a potential photocatalyst or not. To facilitate the water redox reactions, the conduction band edge should be higher than water reduction potentials, whereas the valence band edge should be lower than water oxidation potential. The positions of VBM and CBM of the $\hbox {BeN}_{{2}}$ monolayer has here been determined by the bandgap center energy ($E_{BGC}$) and its bandgap ($E_{g}$) (see Eq. (2)).

3 $$\begin{aligned} E_{CBM/VBM} = E_{BGC} \pm \frac{1}{2} E_{g} \end{aligned}$$

Since $E_{BGC}$ has shown to be quite insensitive to the choice of exchange-correlation functional, we have used the less expensive PBE functional. The photoemission/inverse photoemission (PE/IPE) gap calculated with $\hbox {G}_{{0}}\,\hbox {W}_{{0}}$ was then used to obtain the CBM and VBM energy by adding and subtracting half of the PE/IPE gap from the $E_{BGC}$, respectively. The vacuum level was set to zero to align the energy levels for the $\hbox {BeN}_{{2}}$ monolayer.

The band edge potential diagram is plotted in Fig. S3c. From this figure, it is clear that the band edge positions of $\hbox {BeN}_{{2}}$ straddle the water redox potentials at pH 0, so this meets the thermodynamic requirement of an efficient photocatalyst to split water into hydrogen and oxygen. The CBM is located at 0.56 eV higher in energy than the reduction potential of H$^{+}$/$\hbox {H}_{{2}}$ (− 4.44 eV vs. the vacuum level), and the VBM is located at 1.37 eV lower in energy than the oxidation potential of $\hbox {O}_{{2}}$/$\hbox {H}_{{2}}$O (− 5.67 eV vs.the vacuum level). As the VBM is highly stabilized, it improves the hole transfer process to the redox species and this reduces the probability of charge carrier recombination, which in turn enhances the photocatalytic water splitting activity.

To evaluate the ability of electron-hole transfer, we calculated the effective masses of an electron and a hole along the $\Gamma$-K and $\Gamma$-X directions. Effective of electron and hole were derived from the band structure plot (see Fig. S3a) using the band fitting method. The second-order partial derivative of the valence band maxima and conduction band minima were calculated to obtain the hole effective mass and the electron effective mass, respectively. The calculated effective mass of an electron along $\Gamma$-K and $\Gamma$-X directions are 0.24 $\hbox {m}_{{e}}$ and 0.29 $\hbox {m}_{{e}}$, respectively. Moreover, the calculated effective mass of a heavy-hole along the $\Gamma$-K and $\Gamma$-X directions are 1.81 $\hbox {m}_{{e}}$ and 1.90 $\hbox {m}_{{e}}$, whereas the effective mass of a light-hole along the $\Gamma$-K and $\Gamma$-X directions are 0.27 $\hbox {m}_{{e}}$ and 0.34 $\hbox {m}_{{e}}$, respectively. In order to have an excellent charge carrier mobility, the effective mass of charge carriers should be lower than 0.5 $\hbox {m}_{{e}}$, at least in one crystallographic direction^41^. In the $\hbox {BeN}_{{2}}$ monolayer, the effective masses of both the electron and light-hole along the $\Gamma$-K and $\Gamma$-X directions, are lower than 0.5 $\hbox {m}_{{e}}$. This indicates that the charge carriers would respond quickly, which results in improved carrier mobility. It is interesting to note that the more stabilized valence band edge, in addition to the lower effective mass of holes, will make the $\hbox {BeN}_{{2}}$ monolayer an excellent candidate for photocatalyst for water splitting.

### Oxygen evolution reaction (OER)

The oxygen evolution capabilities of $\hbox {BeN}_{{2}}$ has also been studied by using the calculated reaction free energies of all the possible intermediates. This particular half-reaction is more demanding as it involves four redox processes over a narrow potential range. It also involves the coupling of multiple protons and electrons transfers, and the formation of two oxygen−oxygen bonds. The OER reaction steps can be seen in Eqs. (3)–(6)^42,43^:

4 $$\begin{aligned}&* + H_{2}O \rightarrow *OH + (H^{+}+e^{-}) \end{aligned}$$

5 $$\begin{aligned}&*OH \rightarrow *O + (H^{+}+e^{-}) \end{aligned}$$

6 $$\begin{aligned}&*O + H_{2}O \rightarrow *OOH + (H^{+}+e^{-}) \end{aligned}$$

7 $$\begin{aligned}&*OOH \rightarrow * + O_{2} + (H^{+}+e^{-}) \end{aligned}$$

where *X refers to species X (X = *OH, *O, and *OOH) adsorbed on the $\hbox {BeN}_{{2}}$ surface. We have considered all the possible adsorption sites for the OER intermediates on the $\hbox {BeN}_{{2}}$ surface and the site with the most negative adsorption energy are referred to as the most stable site, and that has been used for further analysis.

The water molecule adsorption on a $\hbox {BeN}_{{2}}$ surface is the very first step of an OER, and it is the fundamental requisite to start the reaction process. The first elementary reaction step of a water molecule adsorbed onto the $\hbox {BeN}_{{2}}$ surface is to convert to *OH (the *notation means that the species is adsorbed to the surface) by releasing one proton-electron pair (H$^{+}$+e$^{-}$). As can be seen from Fig. 3a, the *OH molecule binds chemically onto the Be atom, with the adsorption energy of − 1.34 eV. The free energy change for this reaction step (3) was calculated to be + 2.05 eV. The next reaction step for the adsorbed OH molecule is to convert to *O by releasing another proton-electron pair. In this case, the oxygen atom breaks the Be−N bond and forms a chemical bond with both Be and N. The adsorption energy was found to be − 2.42 eV. The free energy change for this reaction step (4) was calculated to be + 0.58 eV. The third reaction step involves a second water molecule that interacts with the adsorbed O on the $\hbox {BeN}_{{2}}$ surface, forming an *OOH intermediate upon releasing the third electron-proton pair. The *OOH prefers to bind onto the Be atom with the adsorption energy of − 0.61 eV. The free energy change for this reaction process (5) was calculated to be + 2.06 eV. The final reaction in the OER mechanism is the adsorbed OOH onto the $\hbox {BeN}_{{2}}$ surface, which will release the oxygen gas by releasing the fourth proton-electron pair. The calculated free energy change for this reaction (6) was calculated to be − 0.24 eV.

**Figure 4:**
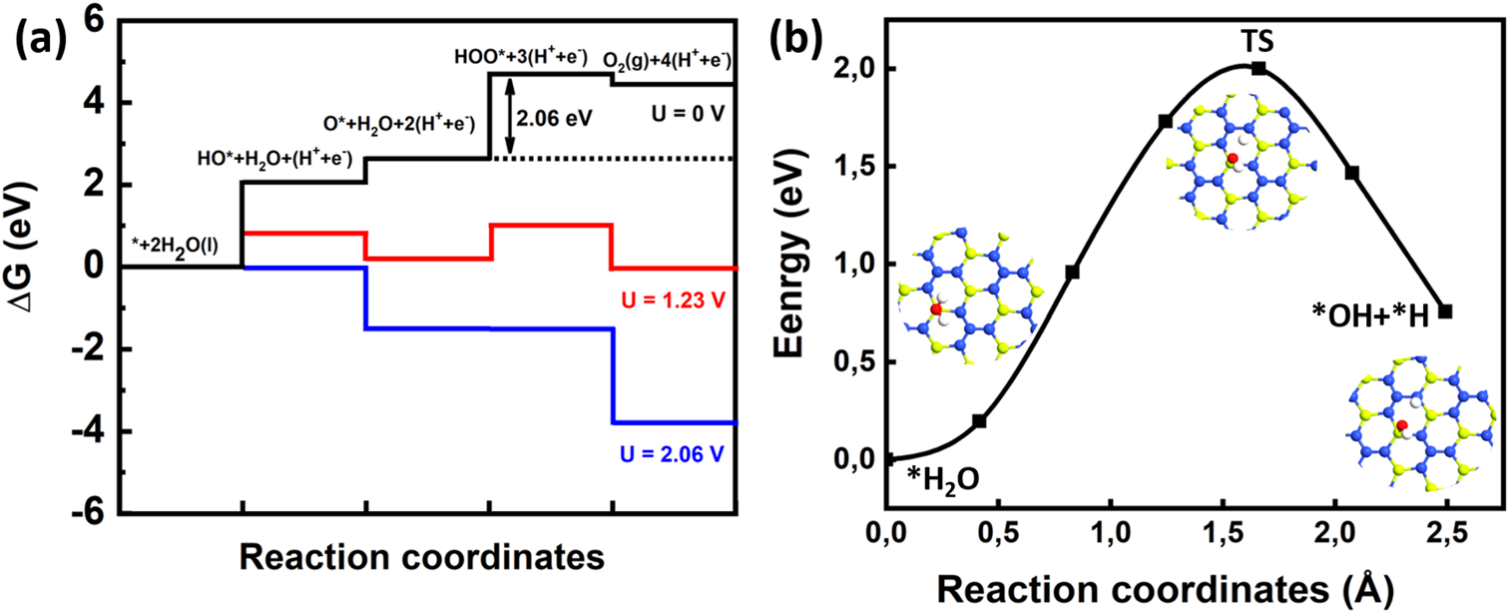
(**a**) Calculated free energy changes for the different intermediated reaction steps involved in the oxygen evolution reaction. (**b**) Minimum energy path for the dissociation of a water molecule into adsorbed OH and H species on a $\hbox {BeN}_{{2}}$ monolayer.

The calculated reaction free energy profile, with different electrode potentials, is displayed in Fig. 4a. As can be seen in figure, all reaction steps are endergonic, with an exception of the final step, $\hbox {O}_{{2}}$ evolution, at U = 0 V (with a total free energy change of + 4.45 eV). The third reaction step, OOH* formation, is the rate-limiting step with a + 2.06 eV free energy change. At the equilibrium potential of 1.23 eV, two reaction steps were found to be endergonic, and two steps were exergonic. The *OH and *OOH formation reaction steps, where the proton is extracted from water, were uphill, whereas the O* and $\hbox {O}_{{2}}$ evolution reaction steps were downhill in nature. The free energy profile at U=2.06 V showed that all reaction steps were exergonic. Hence, the calculated OER overpotential ($\eta$ $^{OER}$) became 0.83 V for $\hbox {BeN}_{{2}}$ monolayer. The holes in the valence band, with a potential of 2.06 eV and above, can thereby oxidize water to oxygen. Furthermore, the band edge alignment resulted in a location of the valence band edge at 2.60 eV, with respect to NHE. Therefore, the holes in the valence band of $\hbox {BeN}_{{2}}$ can oxidize water without any co-catalyst.

The first and third steps in the OER mechanism involves a proton extraction from water, and these two steps are more energy demanding than the other reaction steps. In order to estimate their associated barrier energies, the minimum energy path (MEP) for the dissociation of a water molecule into hydroxide and a proton were identified (i.e., where the proton becomes adsorbed onto $\hbox {BeN}_{{2}}$ surface instead to being transferred to the water medium) (see Eq. (7)).

8 $$\begin{aligned} *H_{2}O \rightarrow *OH + *H \end{aligned}.$$

Figure 4b shows the MEP for the dissociation of a water molecule on the $\hbox {BeN}_{{2}}$ surface, along with the geometries of initial, final, and transition states. The calculated energy barrier for this dissociation reaction is 2 eV, which is lower than the free energy barriers of the first and third reaction steps in OER. Hence, the energy barrier does not affect the OER overpotential. It is very interesting that many photocatalysts can only satisfy HER, but not OER. However, here the presented results show that the $\hbox {BeN}_{{2}}$ monolayer is a promising catalyst for OER.

### Hydrogen evolution reaction (HER)

**Figure 5:**
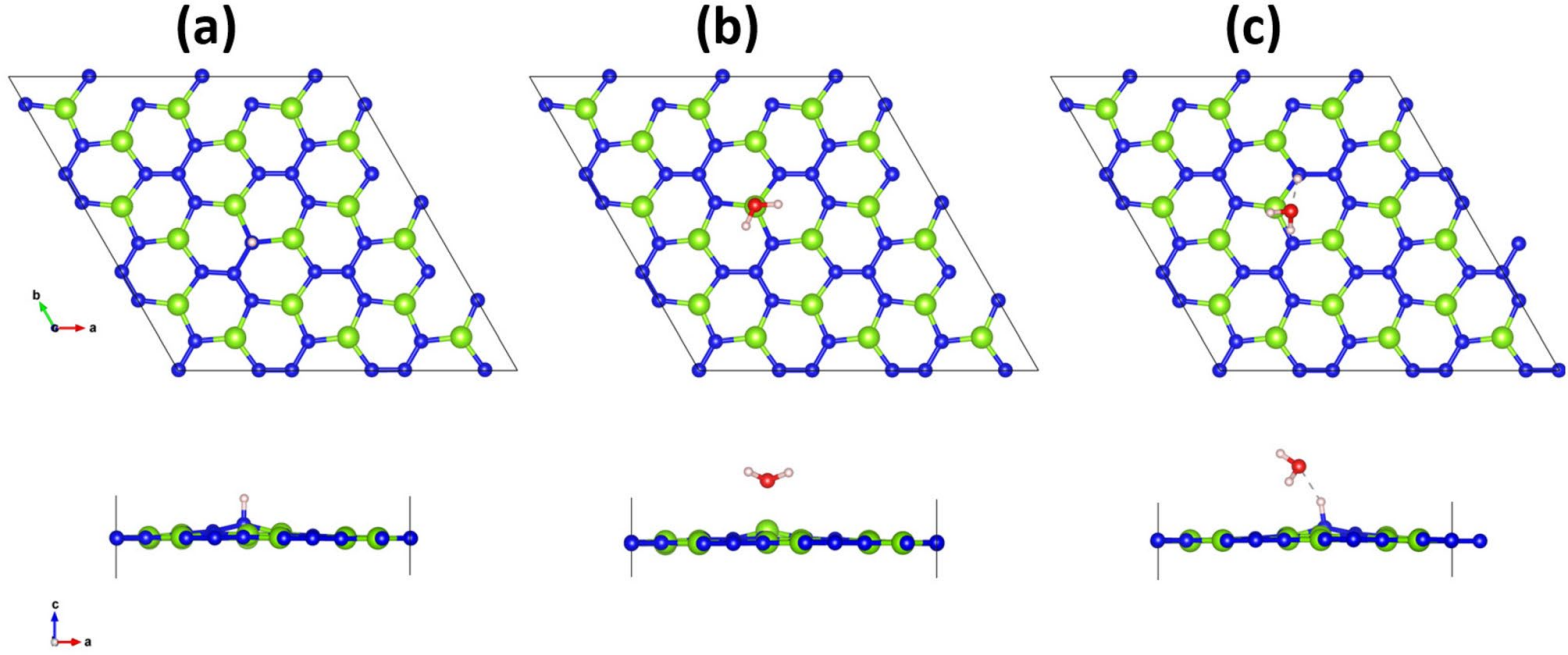
Optimized geometries of possible intermediates in the hydrogen evolution reaction; (**a**) *H, (**b**) *$\hbox {H}_{{2}}$O, and (**c**) *$\hbox {H}_{{3}}$O.

The hydrogen evolution reaction mechanism on the $\hbox {BeN}_{{2}}$ monolayer, has also been studied in the present investigation. Earlier studies have shown that the Gibbs free energy of hydrogen adsorption ($\Delta$G$^{o}_{H}$) is the most important descriptor of HER activity^44,45^. If $\Delta$G$^{o}_{H}$ is close to zero, then the material will be a suitable catalyst for hydrogen evolution. The lower $\Delta$G$^{o}_{H}$ will results in a slow hydrogen release step (because of the strong bonds with hydrogen atoms), whereas higher $\Delta$G$^{o}_{H}$ will make the proton/electron-transfer step endothermal.

**Figure 6:**
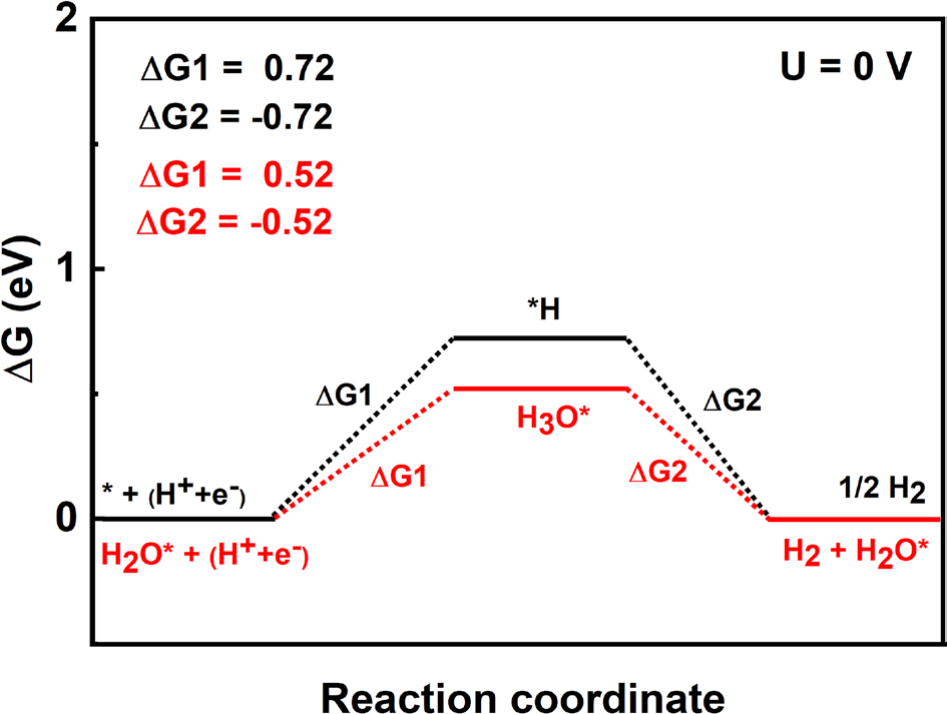
Calculated free energy changes of two suggested hydrogen evolution reaction; via H* formation (in black), or $\hbox {H}_{{3}}$O* formation (in red).

This reaction can proceed in two possible ways, either by hydrogen or by a water molecule. Both situations have here been looked at. The HER reaction steps are as follows (see Eqs. (8)–(11))^46–48^:

9 $$\begin{aligned}&* + (H^{+}+e^{-}) \rightarrow *H \end{aligned}$$

10 $$\begin{aligned}&*H + (H^{+}+e^{-}) \rightarrow H_{2} + * \end{aligned}$$

HER can also proceed from the $\hbox {H}_{{2}}$O molecule

11 $$\begin{aligned}&*H_{2}O + (H^{+}+e^{-}) \rightarrow *H_{3}O \end{aligned}$$

12 $$\begin{aligned}&*H_{3}O + (H^{+}+e^{-}) \rightarrow *H_{2}O + H_{2} \end{aligned}$$

Figure 5a–c show the relaxed structures of HER intermediates *H, *$\hbox {H}_{{2}}$O, and *$\hbox {H}_{{3}}$O, respectively. It can be seen from this Figure that *H prefers to chemically bind on top of N atoms with the adsorption energy of 0.34 eV. The $\hbox {H}_{{2}}$O molecule prefers to physisorb on top of Be atom with an adsorption energy of − 0.24 eV. In the case of *$\hbox {H}_{{3}}$O, it was found to break into a water molecule and H. The adsorption energy was found to be − 0.075 eV. The calculated free energy profile is plotted in Fig. 6. It can be seen from this Figure that the adsorbed H requires higher reaction free energy than $\hbox {H}_{{3}}$O$^{+}$ adsorbate. Hence, the HER for the $\hbox {BeN}_{{2}}$ monolayer will proceed via $\hbox {H}_{{3}}$O$^{+}$ pathway instead of H pathway (in other words, it proceeds via an extra water molecule pathway). The calculated free energy is + 0.72 eV, which indicates that the overpotential for an overall $\hbox {H}_{{2}}$ evolution is − 0.72 V. When an extra water molecule is present, the free energy will decrease to + 0.52 eV, and the overpotential would thereby become − 0.52 V. From the band edge alignment, it was observed that the conduction band edge is located at 0.56 eV above the hydrogen reduction level. It is interesting to note that hydrogen evolution on the $\hbox {BeN}_{{2}}$ monolayer also proceeds without the aid of any co-catalyst.

Though OER and HER can spontaneously occur upon photoexcitation of the $\hbox {BeN}_{{2}}$ monolayer, the respective overpotential is not on par with some of the ideal OER and HER catalysts, like $\hbox {RuO}_{{2}}$^49,50^, $\hbox {IrO}_{{2}}$^51^, and Pt^52^, etc. However, the $\hbox {BeN}_{{2}}$ monolayer is much better than other well-known catalysts, such as g-$\hbox {C}_{{3}}\,\hbox {N}_{{4}}$^53^ (whose OER overpotential is very high). This material can only facilitate HER. g-CN is another example^54^, which shows good activity for OER (but not HER). In addition, the CdS nanotube^55^ has larger HER and OER overpotentials (as compared with the $\hbox {BeN}_{{2}}$ monolayer). The present state-of-the-art photocatalysts uses various surface engineering strategies or Z-scheme processes. The highest reported STH efficiency is 2.0% for a carbon dot loaded $\hbox {C}_{{3}}\,\hbox {N}_{{4}}$ (CDots-$\hbox {C}_{{3}}\,\hbox {N}_{{4}}$) photocatalyst^56^. This is a significant step in achieving the 5% STH target set by the US Department of Energy for photocatalysts in one-step water splitting.

Here, we compare the theoretically calculated band edge positions of $\hbox {BeN}_{{2}}$ with the experimentally measured band edge positions of CDots-$\hbox {C}_{{3}}\,\hbox {N}_{{4}}$. The CBM and VBM of CDots-$\hbox {C}_{{3}}\,\hbox {N}_{{4}}$ is positioned at 0.25 eV higher in energy than the reduction potential of H$^{+}$/$\hbox {H}_{{2}}$ (− 4.44 eV vs. the vacuum level), and 1.29 eV lower in energy than the oxidation potential of $\hbox {O}_{{2}}$/$\hbox {H}_{{2}}$O (− 5.67 eV vs. the vacuum level), respectively. In the case of $\hbox {BeN}_{{2}}$, the CBM is located at 0.56 eV higher in energy than the reduction potential, and the VBM is located at 1.37 eV lower in energy than the oxidation potential, respectively. The VBM position of $\hbox {BeN}_{{2}}$ is comparable with that of CDots-$\hbox {C}_{{3}}\,\hbox {N}_{{4}}$’s VBM, whereas CBM of $\hbox {BeN}_{{2}}$ is 0.25 eV higher than that of CDots-$\hbox {C}_{{3}}\,\hbox {N}_{{4}}$’s CBM. In order to improve the photocatalytic activity of $\hbox {BeN}_{{2}}$, the CBM level should be shifted downward while slightly lowering or stabilizing the VBM. Also, the Gibbs free energy of HER and OER intermediates should be lowered.

In general, the adsorption strengths of the reaction intermediates (OH*, O*, and OOH*) strongly affect the catalytic activities. The adsorption strength should not be too strong, or too weak. Usually, in OER, the rate-determining step would be O* $\rightarrow$ OOH*. This is also the case for the present $\hbox {BeN}_{{2}}$ system, and this is due to the larger adsorption energy of O* (−2.42 eV), as compared with OH* (−1.34 eV) and OOH* (−0.61 eV). The O atom strongly binds with Be and N atoms, which results in too strong O* adsorption. As the rate-limiting step in OER and HER is O* $\rightarrow$ OOH* and H*, one has to alter the electronic properties of the $\hbox {BeN}_{{2}}$ monolayer to weaken the adsorption strength of O* and H* (and thus tune the OER and HER activities, respectively). Therefore, chemical modification such as heteroatom doping, will introduce different kinds of defects. Oxidation, with the purpose to reduce the reaction overpotentials, will be required to significantly improve the electrocatalytic performances Research in this direction is in progress.

## Conclusions

The reaction mechanism of the photocatalytic water splitting onto a $\hbox {BeN}_{{2}}$ monolayer has here been theoretically investigated by using density functional calculations. The calculated effective mass of electron and the hole indicate an excellent mobility in the lattice. This is a circumstance that improves the charge carrier separation, and, thus, reduces the recombination rate. In OER, it was found that the reaction step involving *OOH formation, was the rate-limiting step. In addition, the HER would proceed via the *$\hbox {H}_{{3}}$O pathway. The calculated overpotential for oxygen evolution and hydrogen evolution reaction is 0.83 V and 0.52 V (with reference to NHE), respectively. From band alignment with respect to water redox potentials, the results indicate that the photo-generated holes at the valence band can oxidize water to oxygen, and the photo-generated electrons at the conduction band can reduce water to hydrogen (without the aid of any co-catalyst). Hence, these results demonstrate that $\hbox {BeN}_{{2}}$ monolayer will act as a potential bifunctional catalyst that can reduce and oxidize water in the production of hydrogen.

## Methods

All calculations were performed using the projector augmented wave (PAW) method, implemented in the *Vienna Ab initio Simulation Package* (VASP)^57^. The generalized gradient approximation proposed by Perdew, Burke, and Ernzrhof (GGA-PBE)^58^ with van der Waals(vdW) correction proposed by Grimme, was used for the geometrical relaxation (due to its good description of long-range vdW interactions)^59^. Moreover, the Heyd-Scuseria-Ernzerhof (HSE06) hybrid functional was used in calculating the electronic structures (owing to the underestimation of semiconductor bandgaps when using the PBE functional)^60^. The Brillouin zone was sampled by using the Monkhorst-Pack scheme, with an employed **k**-mesh of $5 \times 5\,\times$1^61^. In addition, a plane-wave basis set, with a cutoff energy of 450 eV was used. A large vacuum of 18 Å was used to avoid spurious interactions. All structures were optimized until the total energy converged to less than 10$^{-5}$ per atom, and the Helman-Feynman forces (that acting on each atom) was less than 0.01 eV/Å  upon ionic relaxation. The postprocessing of the calculated data (using VASP) was done by using the VASPKIT code^62^. Moreover, the PHONOPY code was employed to calculate the phonon dispersion curves of the $\hbox {BeN}_{{2}}$ monolayer^63^. In addition, $ab-initio$ molecular dynamics (AIMD) simulations were used to evaluate the stability of the $\hbox {BeN}_{{2}}$ monolayer at 300 K. When studying the $\hbox {BeN}_{{2}}$ interface in vacuum, the simulations lasted for 10 ps, with a time step of 1 fs. For the $\hbox {BeN}_{{2}}$/water interface simulations, they lasted for 5 ps (with a time step of 0.5 fs). The Nosé algorithm^64^ was used to control the temperature. The transition state was searched using the climbing image nudged elastic band (CI-NEB) method^65,66^, as implemented in QuantumATK-2019.03.

Free energies for the intermediates in the oxygen evolution reaction (OER) and hydrogen evolution reaction (HER) have been computed by using the computational hydrogen electrode (CHE) model developed by Nørskov^46,47,67^ and co-workers. By using this model, free energy changes of electrochemical reactions have been calculated by using the expression shown in Eq. (12)

13 $$\begin{aligned} \Delta {G} = \Delta {E} + \Delta {ZPE} - T \Delta {S} + \Delta {G_U} + \Delta {G_{pH}} \end{aligned}$$

where $\Delta {E}$, $\Delta {ZPE}$, and $\Delta {S}$ are the difference in DFT calculated total energy, zero-point energy, and entropy between final and initial states, respectively. The $\Delta {G_U}$ = −*e*U, where U is the potential of the photogenerated electrons/holes with respect to the normal hydrogen electrode (NHE), and *e* is the number of transferred electrons. Here, $\Delta {G_{pH}} = 2.303\,{k}_{B}$T pH, where *k*B is the Boltzmann constant, the temperature T has been taken as 300 K, and pH=0. Zero-point energy correction and entropies for adsorbed intermediates were calculated from vibrational frequencies analysis using density functional perturbation theory, whereas the entropies of the species in the gas phase were taken from the NIST database^68^.

## Supplementary information

Supplementary Figures.
