# Supplementary material for: Theoretical investigation on BeN2 monolayer for an efficient bifunctional water splitting catalyst
Source: Sci Rep. 2020 Dec 8;10:21411. doi: 10.1038/s41598-020-77999-8 (PMC7722721; doi:10.1038/s41598-020-77999-8)
Supplement: Supplementary file 1 — Supplementary Figures. [file 41598_2020_77999_MOESM1_ESM.pdf]

Supplementary Information

Theoretical Investigation on BeN_2 Monolayer for an Efficient Bifunctional Water Splitting Catalyst

M.R. Ashwin Kishore,[1] R. Varunaa,[2] Amirhossein Bayani,[1] and Karin Larsson[1,*]
[1]Department of Chemistry, Ångström Laboratory, Uppsala University,
Uppsala, Box 538 751 21, Sweden
[2]Department of Physics, Central University of Tamil Nadu,
Thiruvarur, 610101, India

August 29, 2020

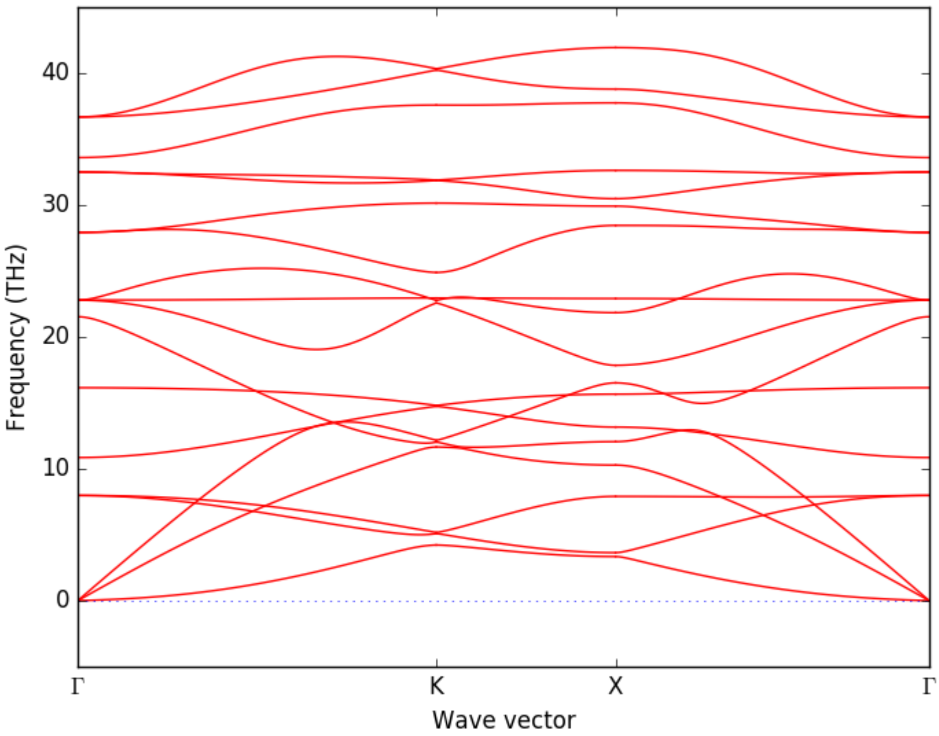

Figure S1: Dispersion of phonon modes of the BeN_2 monolayer calculated with 3×3×1 supercell.

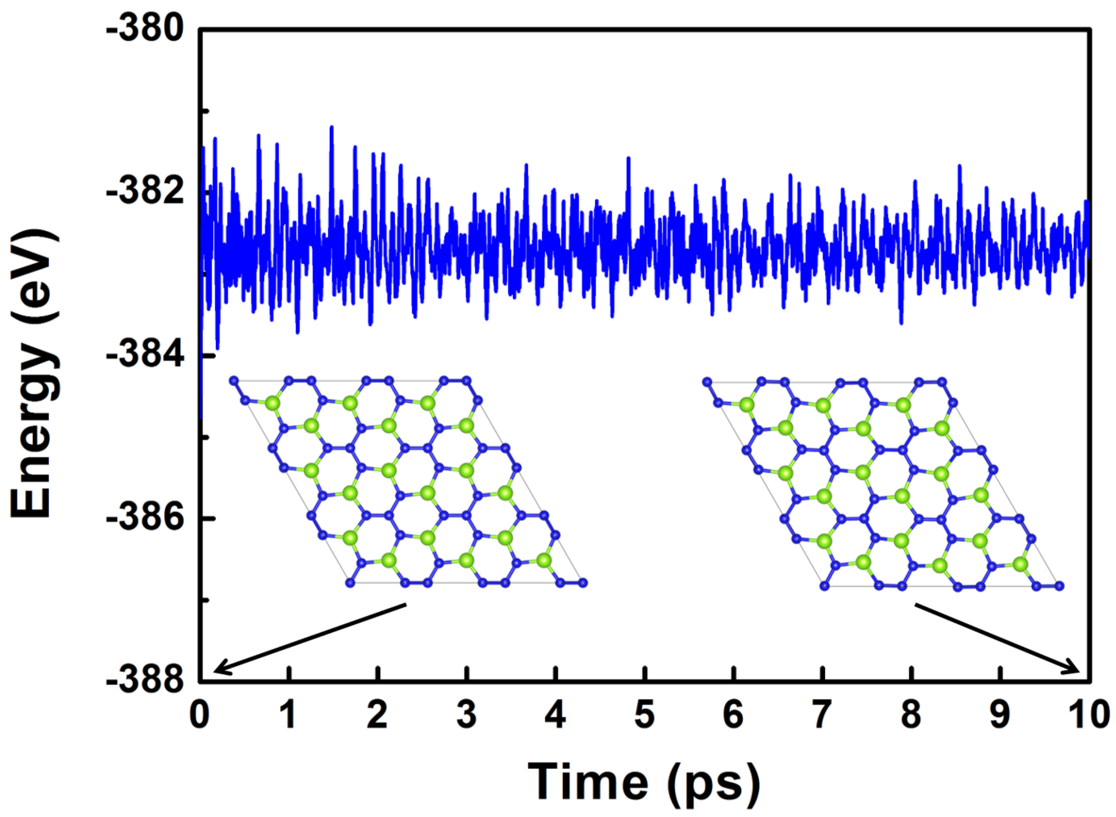

Figure S2: Total energy fluctuation during AIMD simulations at 300 K and the insets are snapshot structures of the BeN_2 monolayer at 0 ps and 10 ps.

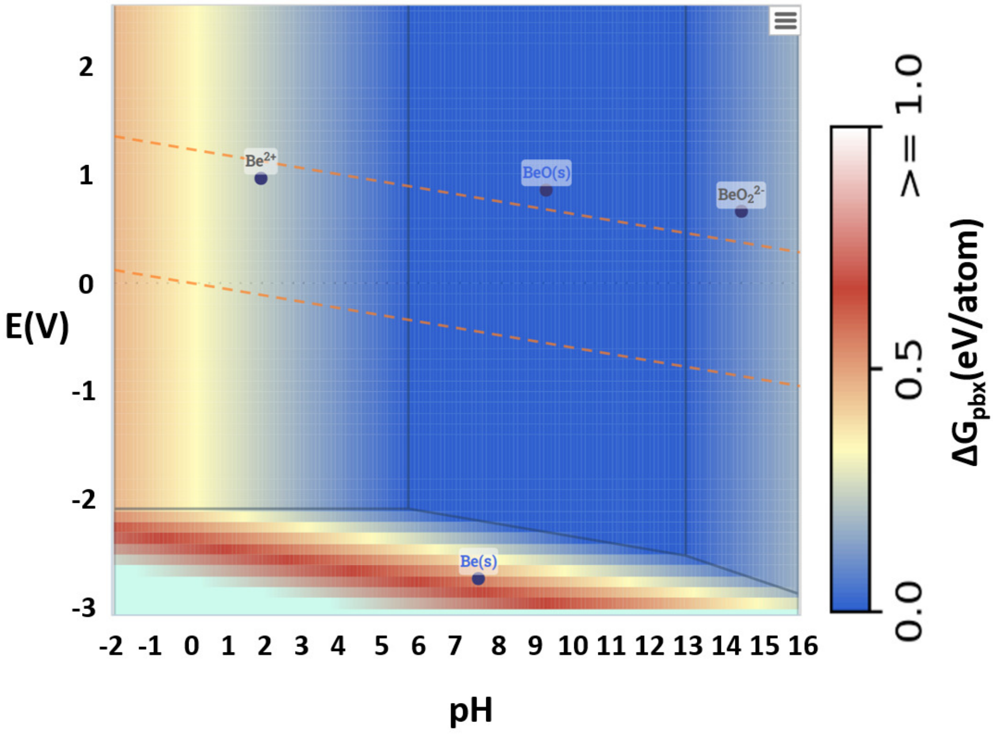

Figure S3: Computationally predicted Pourbaix diagram of BeN_2 system. The stable phases include ion phase Be(s) and BeO(s). The orange dashed lines denote potentials of 0 V vs RHE and 1.23 V vs RHE.

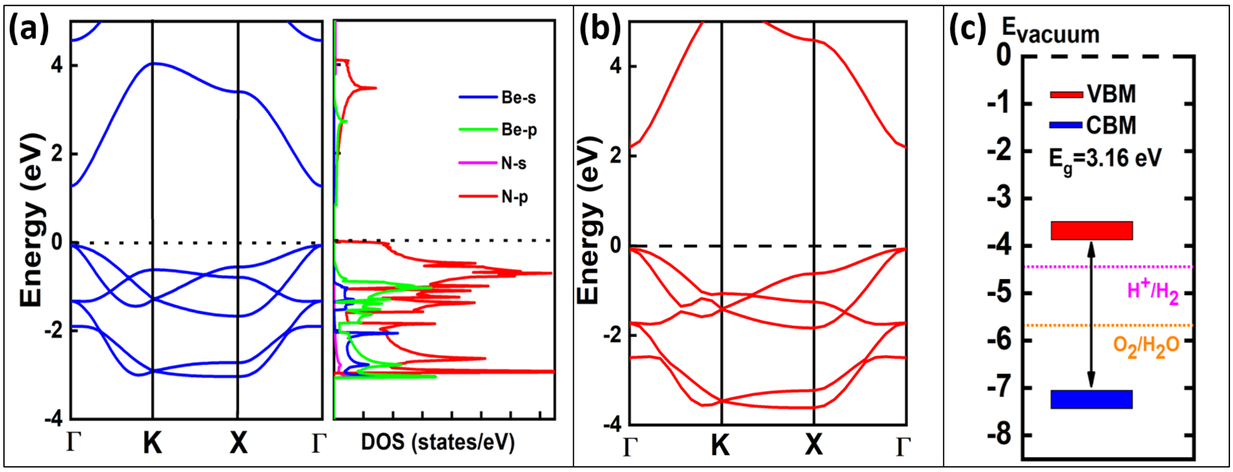

Figure S4: Calculated band structure and density of states of BeN_2 monolayer obtained from PBE and band structure using HSE06 functional are shown in (a) and (b), respectively. The Fermi level is set to zero. (c) Calculated band edge positions of BeN_2 monolayer with respect to vacuum potential. The dashed lines are water redox potentials at pH = 0

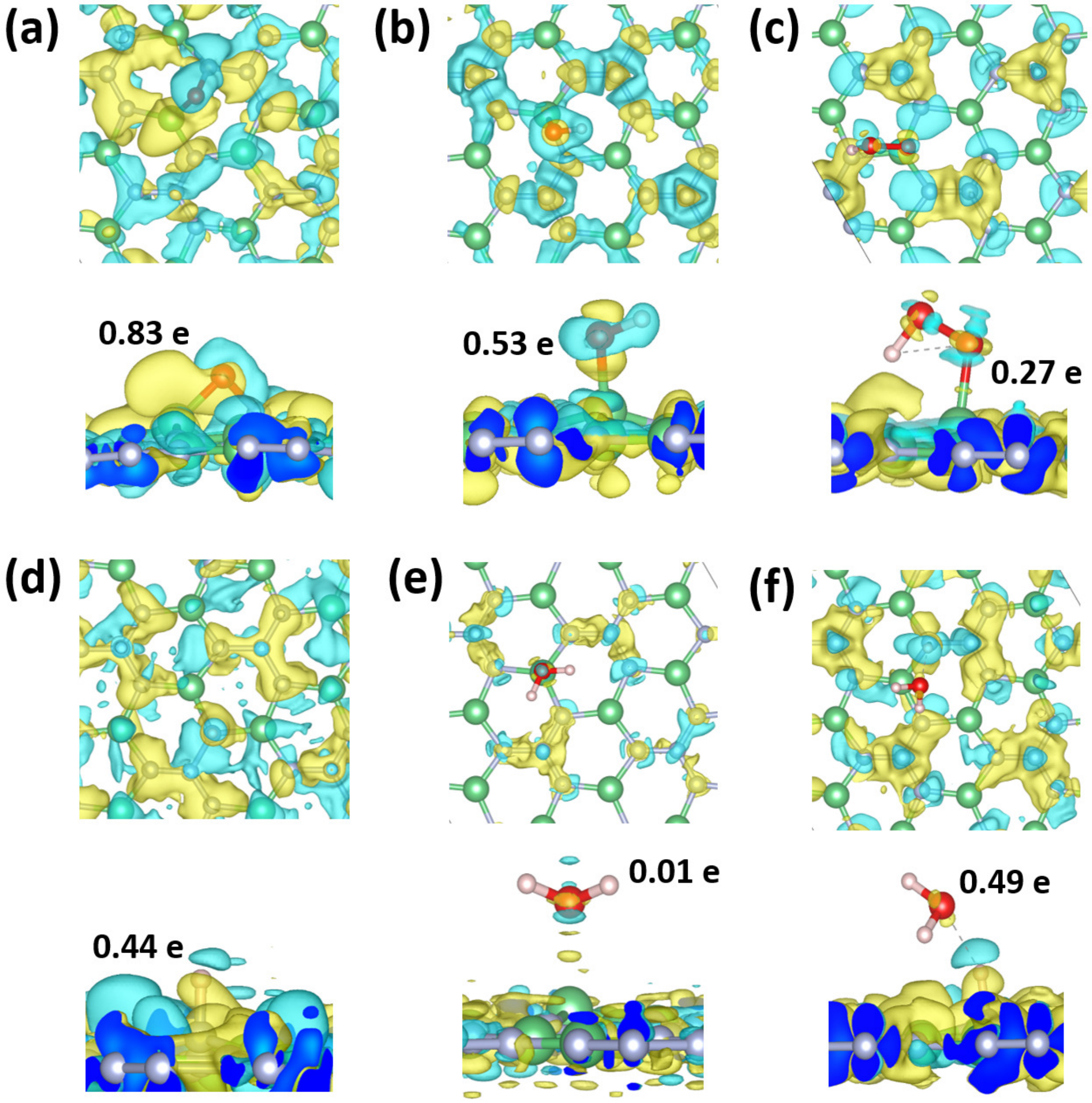

Figure S5: Iso-surface plot of charge density difference between BeN_2 monolayer and reaction intermediates (a) *O, (b) *OH, (c) *OOH, (d) *H, (e) *H_2O, and (f) *H_3O. The isosurface value is set to be 0.015 Å^{-3}, The color yellow (cyan) represents an accumulation (loss) of electrons.
